# Transcriptomic and proteomic profiling of pulmonary mucormycosis reveals a failed activation of host immune response

**DOI:** 10.3389/fimmu.2026.1732782

**Published:** 2026-02-13

**Authors:** Jinjin Zhong, Xiaomin Cai, Yuchen Cai, Tingting Zhao, Die Hu, Chao Sun, Yueyan Ni, Yu Gu, Xin Su

**Affiliations:** 1 Department of Respiratory and Critical Medicine, Jinling Hospital, Affiliated Hospital of Medical School, Nanjing University, Nanjing, China; 2 Department of Respiratory and Critical Medicine, Nanjing Drum Tower Hospital, Affiliated Hospital of Medical School, Nanjing University, Nanjing, China

**Keywords:** pulmonary mucormycosis, mucormycosis, immune response, transcriptomics, proteomics

## Abstract

**Introduction:**

Mucormycosis is one of the most life-threatening fungal infections with delayed diagnosis and limited antifungal treatments. The transcriptome and proteome of pulmonary mucormycosis have not been fully investigated.

**Methods:**

We obtained lung tissues and paired controls from five pulmonary mucormycosis patients and utilized transcriptomic and proteomic approaches to explore host immune response during pulmonary mucormycosis.

**Results:**

Our transcriptomic analysis found a number of up-regulated genes and pathways associated with immune defense. These genes were related to iron metabolism, pattern recognition receptors (PRRs), cytokines, chemokines et al., which enriched in pathways involved in both innate and adaptive immunity. However, proteomic profiling revealed limited upregulation of immune-related proteins and global suppression of pathways associated with host defense, especially those related to cell junction and cytoskeletal dynamics, indicating a failed activation of host immune response.

**Discussion:**

Given the findings of compromised immune function at infection sites, enhancing adjuvant immunotherapy and intensifying localized antifungal treatments may be beneficial for this refractory infection. Our study firstly investigated the immune landscape in pulmonary mucormycosis through combined transcriptomic and proteomic profiling, which could provide novel mechanistic insights for the prevention and treatment of pulmonary mucormycosis.

## Introduction

Mucormycosis is one of the most aggressive fungal infections with high mortality and an increase mobility in recent decades, especially during the pandemic of COVID-19, which has become a public health problem (1, 2). The members of the order Mucorales such as *Rhizopus*, *Mucor*, and *Lichtheimia* species are responsible for most cases while *Cunninghamella*, *Apophysomyces*, *Saksenaea*, *Rhizomucor*, *Cokeromyces*, *Actinomucor*, and *Syncephalastrum* cause fewer infections (3). Invasive mucormycosis usually occurs in individuals underlying medical comorbidities or immunosuppression (4, 5). Diabetes is a major risk factor and immunocompromised patients undergoing chemotherapy, cancer immunotherapy, hematopoietic stem-cell transplantations and solid organ transplantations are more susceptible to this infection (4, 5). In addition, glucocorticoid treatment, trauma, iron overload and malnourishment are also associated with the increased incidence of mucormycosis (4, 5).

The forms of mucormycosis mainly include rhino-orbital-cerebral, pulmonary, cutaneous and soft-tissue, gastrointestinal and renal mucormycosis (6, 7). Lungs are the third main location after the rhino-orbito-cerebral areas and skin (8). Pulmonary mucormycosis usually progresses rapidly and carries a high mortality rate (9). Liposomal amphotericin B is first-line therapy of antifungal agents and the step-down treatment includes oral isavuconazole or posaconazole. It is often difficult to cure by using drug therapy alone so surgical intervention is strongly recommended to improve prognosis and it is also critical to correct risk factors (6, 9). Nevertheless, the delayed diagnosis and limited pharmacological treatments result in poor survival rates (9). Therefore, it is necessary to improve our understanding of host immune response during mucormycosis, which can help exploring new approaches for prevention and antifungal therapies.

To date, the pathogenesis and immune mechanisms of pulmonary mucormycosis are not fully understood and remain unexplored. Mucormycosis is characterized by extensive tissue invasion and destruction and is the most angioinvasive of all fungal infections (1). Spores of Mucorales are dispersed by air and taken up by humans via inhalation; once the host immune function is compromised, these inhaled fungi are dreaded to cause fatal disease (10). After Mucorales invasion, the innate immune system works as the first defense line to prevent spore germination, including host barrier cells, alveolar macrophages, neutrophils and natural killer (NK) cells (1, 11). Although adaptive immunity is also activated, there is limited evidence for a critical role of it in combating mucormycosis (11). Host iron acquisition is another important factor for progression of mucormycosis (11). Given the increased incidence and limited therapeutic options, it is warranted to further explore the immune response in pulmonary mucormycosis to shed light on novel therapeutic interventions.

Assessing the transcriptomes and proteome during infection is a straightforward strategy to provide novel insights on the immune landscape of pulmonary mucormycosis. Previous studies have investigated the transcriptomic profile of COVID-19 associated mucormycosis (CAM) and reported that deficient phagocytosis, cellular iron overload and a hyperglycaemic state contribute to the progress of mucormycosis (12, 13). There are also a few researches focused on animal models (fruit fly, mouse and zebrafish models) of mucormycosis and infected cell models such as airway epithelial cells, human peripheral blood mononuclear cells (PBMCs) and macrophages (AMs), which revealed the antifungal immune response from different aspects (14–21). However, there is a paucity of studies on the pulmonary immune response of mucormycosis patients. As for proteomics, there is also a lack of related research. Thus, it is of great value to investigate the transcriptome and proteome of pulmonary mucormycosis.

In this study, we enrolled patients with pulmonary mucormycosis who underwent surgery and aimed to investigate immune landscape of infected lung tissues using transcriptomic and proteomic approaches. Transcriptomic analysis revealed that fungal infection induced a widespread up-regulation of genes associated with immune response and significant enrichment of multiple signaling pathways related to both innate and adaptive immunity. Nevertheless, we observed limited immune-associated proteins increased in proteomic profiling, suggesting compromised host immune function in protein level. Collectively, our results suggest impaired activation of host immune defense during pulmonary mucormycosis. The identification of altered transcripts and proteins could contribute to a better understanding of immune mechanisms and pathophysiological process and provide new perspectives for the prevention and treatment of mucormycosis.

## Methods

### Study population

We enrolled five pulmonary mucormycosis patients who underwent surgery at the Nanjing Jinling Hospital and Nanjing Drum Tower Hospital from September 2022 to March 2024. This study was approved by the Institute Ethics Committee of Nanjing Jinling Hospital and Nanjing Drum Tower Hospital (approval number: 2022DZGZR-114) and performed in accordance with the Declaration of Helsinki. Clinical characteristics of patients are shown in **Table 1**. These patients were diagnosed according to the ECMM and MSG/ERC guidelines (6). All five patients have received adequate systemic antifungal therapy prior to surgery. However, due to the lack of significant clinical improvement and disease progression, surgical resection of the infected lung tissues was performed according to guideline recommendations (6). We obtained the infected lung specimens and the non-infected tissues of corresponding lung lobes as control group for transcriptomics and proteomic analysis. Infected tissues were identified based on intraoperative gross examination of visible lesions, and areas with the most severe involvement were selected. Non-infected controls were obtained from macroscopically normal regions located furthest from the lesion within the same resected lobe. Histopathological examination was performed to confirm the presence of the fungal hyphae consistent with Mucorales (**Supplementary Figure S1**). A subset of the specimens was obtained from multiple infected lobes of one patient. Lung tissues were stored in -80°C refrigerator before total RNA and protein extraction.

**Table 1:** Clinical characteristics of the study population.

Characteristics	Mucormycosis (n=5)
Age (years)	52.8 (14.1)
Gender(male/female)	4/1
Comorbidities	
Diabetes	5 (100%)
Ketoacidosis	2 (40%)
Hypertension	3 (60%)
History of smoking	3 (60%)
Pharmacotherapy	5 (100%)
Outcome (Died in hospital)	1 (20%)

### RNA extraction, library preparation, sequencing and bioinformatics analysis

We performed transcriptomic profiling on seven paired lung tissue specimens obtained from five patients. Total RNA was extracted using Trizol reagent and then we assessed the quality using agarose gel electrophoresis and the Agilent 2100 Bioanalyzer system. One infected lung specimen was excluded from subsequent analyses due to excessive RNA degradation. Then we used Oligo(dT) magnetic beads to enrich mRNA with polyA structure from total RNA and interrupted mRNA into 200-300bp fragments. Next, we synthesized the first strand of cDNA using a 6-base random primer and reverse transcriptase. Second-strand cDNA was generated using the first-strand cDNA as template. After library construction, the library fragments were enriched by PCR amplification and then we assessed library quality control using an Agilent 2100 Bioanalyzer. Finally, the library was sequenced on Illumina NovaSeq NGS platforms (Illumina).

After sequencing, the raw data in FASTQ format was generated. Then we performed quality filtering, including removal of reads contaminated by adapters at the 3’ end and elimination of reads with a mean quality score below Q20. Filtered reads were aligned to the human genome (Ensembl GRCh38) using HISAT2 software. Gene-level read counts were generated with HTSeq as raw expression values and expression levels were normalized using FPKM (Fragments Per Kilobase of transcript per Million mapped reads). Differentially expressed genes (DEGs) analysis was conducted by R software with package DESeq2. Given the limited sample size and the inherent heterogeneity of human clinical samples, we defined DEGs by |log2FoldChange| > 1 and raw p value < 0.05 for exploratory transcriptomic profiling, while FDR-adjusted results are provided in **Supplementary Tables 1**, **2** as a sensitivity analysis. PCA was performed on the normalized genome-wide expression matrix to visualize overall transcriptomic variation between two groups. Volcano plots and heatmaps were generated by ggplot2 and pheatmap package and genomeCircos was generated by Circlize package. The over-representation analysis (ORA) of gene ontology (GO) enrichment and Kyoto Encyclopedia of Genes and Genomes (KEGG) enrichment were performed by topGO and clusterProfiler package, respectively, based on the candidate DEG list. To avoid spurious enrichment driven by very small gene sets, enriched terms/pathways containing fewer than 3 genes were excluded. Enrichment results are reported with both raw p-values and FDR-adjusted values. Gene set enrichment analysis (GSEA) was performed using the GSEA software (Broad Institute) with KEGG gene sets from MSigDB and enrichment results with FDR q < 0.25 and |NES| > 1 were considered significant. Raw data of transcriptomic profiling has been deposited in in the European Nucleotide Archive (ENA) under accession number Project PRJEB101444.

### Protein extraction, LC-MS/MS and bioinformatics analysis

We performed proteomic profiling on four paired lung tissue specimens from three patients. The protein was extracted by adding SDT lysate to the lung tissue, followed by boiling water bath, ultrasonic fragmentation, and centrifugation at 16000g for 20min at 4 °C. Then we collected the resulting supernatant and quantified the protein using the bicinchoninic acid (BCA) assay. The quality of extracted protein was assessed by sodium dodecyl sulfate-polyacrylamide gel electrophoresis (SDS-PAGE). Next protein was subjected to filter-aided sample preparation (FASP) for tryptic digestion and the resultant peptides were desalted using C18 solid-phase extraction cartridges, vacuum-concentrated, and reconstituted in 0.1% formic acid (FA) for quantification prior to liquid chromatography-mass spectrometry (LC-MS) analysis. Finally, peptide chromatographic separation was performed using a Vanquish Neo UHPLC system (Thermo Scientific), followed by data-independent acquisition (DIA) mass spectrometry analysis on an Orbitrap Astral mass spectrometer (Thermo Scientific).

Raw mass spectrometry files were analyzed using DIA-NN software and searching against the human Uniprot database (version 07/2023). Proteins and peptides were filtered at a false discovery rate (FDR) threshold of 1% (Q-value ≤ 0.01) to generate the final dataset. Considering the incomplete proteome coverage and the wide dynamic range of protein abundance, we defined differentially expressed proteins (DEPs) by |log2FoldChange| > 0.585 and p value < 0.05. DEPs analysis was performed by R software with package DESeq2. Volcano plots and heatmaps were generated by ggplots2 and pheatmap package. The GO and KEGG enrichment were performed by clusterProfiler package and enriched terms/pathways containing fewer than 3 proteins were excluded. Enrichment results are reported with both raw p-values and FDR-adjusted values. The Venn diagram was generated using the online bioinformatics analysis platform (https://www.bioinformatics.com.cn) and the UpSet plot was generated by UpSetR package. Raw data of proteomic profiling has been deposited to ProteomeXchange Consortium (iPROX) repository with the accession number IPX0013889000.

## Results

### Clinical characteristics of participants

We enrolled five hospitalized pulmonary mucormycosis patients who underwent surgical procedures from September 2022 to March 2024 at Nanjing Jinling Hospital and Nanjing Drum Tower Hospital. **Table 1** summarizes the main characteristics of the participants. All of five patients had diabetes mellitus, which might constitute a potential factor for their infection. Among them, two patients had ketoacidosis before being diagnosed with pulmonary mucormycosis. All patients had a proven diagnosis by histopathological examination of bronchoscopic biopsy or surgical resection specimens and received antifungal drug treatment, but the therapeutic effect is limited. Therefore, further surgical treatment was carried out. Ultimately, one patient had fatal clinical outcome.

### Transcriptomic profiling in response to pulmonary mucormycosis

**Figure 1:**
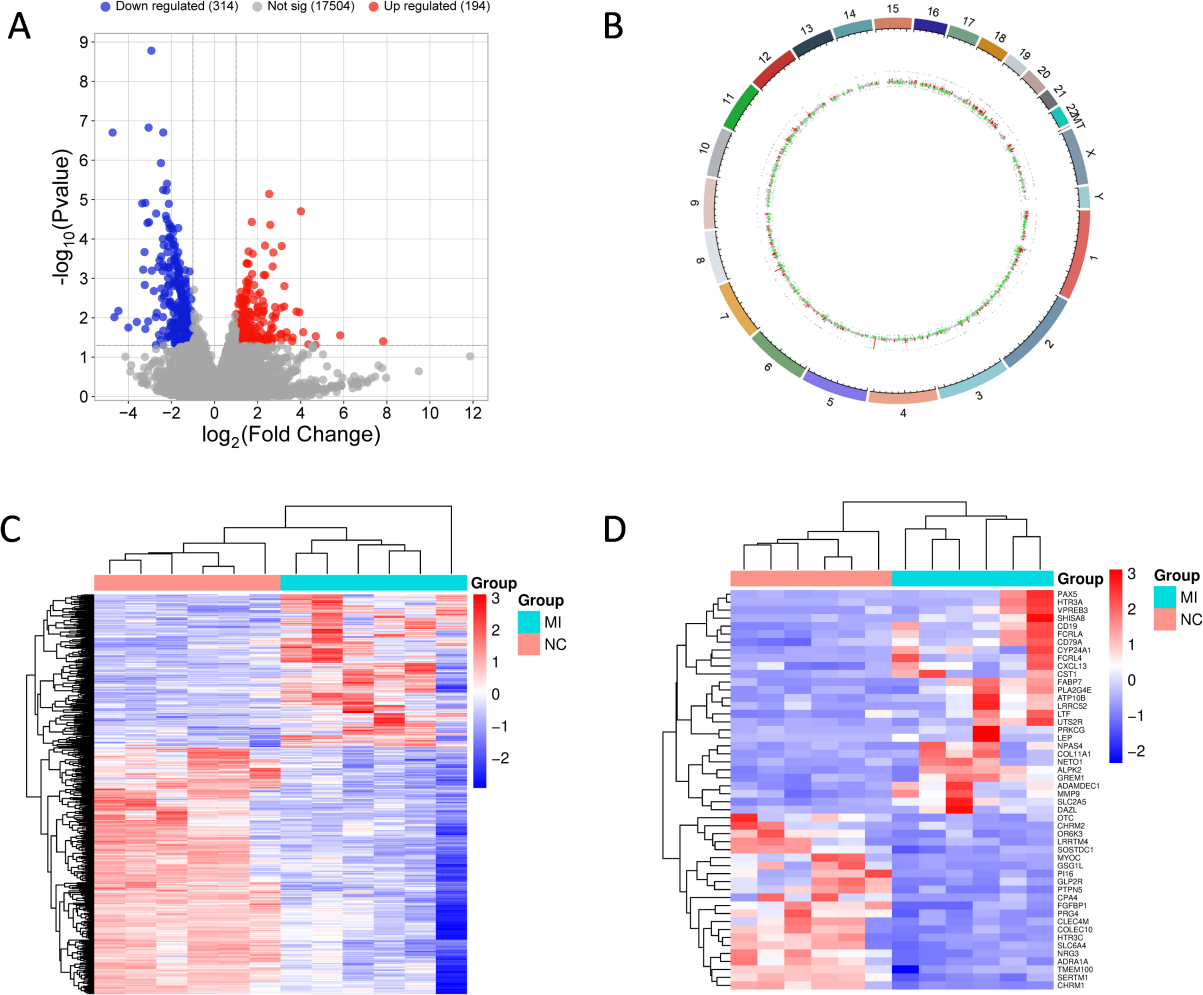
Transcriptional profiling in response to pulmonary mucormycosis. **(A)** Volcano plots of transcripts in infected lung tissues and non-infected controls. The genes with a p value < 0.05 and |log2FoldChange| > 1 were considered DEGs. Red and blue dots indicate up-regulated and down-regulated DEGs, respectively. Gray dots indicate the non-significant genes. **(B)** Circos plot of DEGs in infected lung tissues and controls. The outermost circle represents the chromosome bands. Red and green bars indicate the log2FC values of up-regulated and down-regulated DEGs, respectively, while the gray dots represent the log2FC values of non-significant genes. **(C, D)** Heatmap of all DEGs **(C)** and top 50 most significantly DEGs **(D)** distribution in infected lung tissues and controls. Columns represent the samples and groups and rows represent the DEGs. The red and blue genes represent a higher and a lower expression, respectively. The color intensity (blue to red) corresponds to their expression Z-scores.

The transcriptional response of pulmonary mucormycosis was assessed using RNA sequencing. Quality control showed excessive RNA degradation in one sample. After its exclusion, we included six paired samples from five patients to perform transcriptomic profiling. We found a total of 508 DEGs (|log2FoldChange| > 1 and raw p value < 0.05) compared to uninfected tissues, including 194 up-regulated genes and 314 down-regulated genes (**Figure 1A**; **Supplementary Tables S1**, **S2**). To further explore the genomic distribution of these DEGs, we generated a circos plot to show that transcriptional alterations were broadly distributed, indicating a global transcriptional response against infection (**Figure 1B**). For hierarchical clustering analysis, the heatmap of all DEGs (**Figure 1C**) and top 50 most significantly altered DEGs (**Figure 1D**) revealed different transcriptomic profiling between infected and control groups. Besides, PCA of the transcriptome showed partial separation between MI and paired NC tissues along PC1, with substantial overlap between groups (**Supplementary Figure S2**).

Of note, the most significantly up-regulated gene, HAMP (Hepcidin Antimicrobial Peptide), encoded hepcidin, which plays an important role in iron metabolism and immune response to fungal infection. Furthermore, other three up-regulated genes were also associated with iron homeostasis, including HP (haptoglobin), TP (transferrin) and LTF (lactotransferrin). As we all know, iron metabolism is important in fungal infection, especially in mucormycosis (22, 23). Therefore, these results confirmed the important role of iron in antifungal immunity.

Among up-regulated DEGs, there were a lot of genes involved in host defense (**Table 2**). For instance, transcripts of several genes encoding PRRs were up-regulated, including CLEC17A (C-Type Lectin Domain Containing 17A), TLR10 (Toll Like Receptor 10), ZBP1(Z-DNA Binding Protein 1) and AIM2 (Absent In Melanoma 2), which might play a role in the recognition of fungal pathogens (**Table 2**).

**Table 2:** Up-regulated DEGs might involve in host defense. .

Gene	Protein	Foldchange (MI/NC)	log2FC	P-value
Iron metabolism				
HAMP	hepcidin antimicrobial peptide	5.8154498	2.5398908	7.202E-06
HP	haptoglobin	3.4891776	1.802887	0.0034991
TF	transferrin	2.6678825	1.4156951	0.0193249
LTF	lactotransferrin	10.124518	3.3397813	0.0233459
PRRs				
TLR10	toll like receptor 10	2.9720411	1.5714541	0.0013128
ZBP1	Z-DNA binding protein 1	2.4893453	1.3157664	0.0050478
CLEC17A	C-type lectin domain containing 17A	4.5821685	2.1960305	0.0086864
AIM2	absent in melanoma 2	3.790524	1.9223973	0.0183123
Chemokines and receptors				
CCL11	C-C motif chemokine ligand 11	2.865274	1.5186731	0.0019372
CCL19	C-C motif chemokine ligand 19	2.7618355	1.4656274	0.0188923
CXCL13	C-X-C motif chemokine ligand 13	12.371225	3.6289165	0.0319037
CCR7	C-C motif chemokine receptor 7	2.38763	1.2555793	0.0083851
Interleukin receptors				
IL2RA	interleukin 2 receptor subunit alpha	2.7484426	1.4586144	0.0042677
IL17REL	interleukin 17 receptor E like	3.4812199	1.7995929	0.0322635
TNF receptor superfamily				
TNFRSF9	TNF receptor superfamily member 9	2.4344722	1.283609	0.0128733
TNFRSF13C	TNF receptor superfamily member 13C	6.1933132	2.6307114	0.0147028
TNFRSF17	TNF receptor superfamily member 17	4.1601326	2.0566295	0.0306632
TNFRSF7 (CD27)	TNF receptor superfamily member 7(CD27 molecule)	2.0537304	1.0382468	0.0199653
Interferon pathway				
IRF4	interferon regulatory factor 4	2.6233495	1.39141	0.0090144
IFITM10	interferon induced transmembrane protein 10	2.477702	1.3090027	0.0237167
IFRD1	interferon related developmental regulator 1	2.0491032	1.0349926	0.0332228
SYNDIG1	synapse differentiation inducing 1	2.4133972	1.2710654	0.0368025
Matrix Metallopeptidase				
MMP9	matrix metallopeptidase 9	8.6127715	3.1064776	0.0366107
Autophagy pathway				
VMP1	vacuole membrane protein 1	2.816066	1.4936811	0.0035877
RUFY4	RUN and FYVE domain containing 4	2.8689834	1.5205396	0.0351346
Fc receptor superfamily				
FCRL4	Fc receptor like 4	57.085189	5.8350446	0.0279528
FCRL5	Fc receptor like 5	5.1169671	2.355289	0.0008334
FCRL2	Fc receptor like 2	2.7183722	1.442743	0.0325785
FCRLA	Fc receptor like A	7.2345617	2.8549056	0.0078346
FCMR	Fc mu receptor	3.159902	1.6598798	0.0119115
Immunoglobulin superfamily				
IGDCC4	immunoglobulin superfamily DCC subclass member 4	3.0153886	1.5923439	0.0010675
SIRPG	signal regulatory protein gamma	2.394184	1.259534	0.008982
SLAMF6	SLAM family member 6	2.2394326	1.1631332	0.0108861
CTLA4	cytotoxic T-lymphocyte associated protein 4	2.1817214	1.1254669	0.0184501
CD19	CD19 molecule	9.4391382	3.2386551	0.0051973
JCHAIN	joining chain of multimeric IgA and IgM	2.338466	1.2255624	0.0022004
IGLL5	immunoglobulin lambda like polypeptide 5	4.3868182	2.1331749	0.0339092
Other cluster of differentiation				
CD72	CD72 molecule	2.2978042	1.2002558	0.0044715
CD79A	CD79a molecule	7.0187016	2.8112042	0.0100814
CD22	CD22 molecule	2.236351	1.1611466	0.0288255
MS4A1	membrane spanning 4-domains A1	6.0126952	2.5880118	4.381E-05
CD28	CD28 molecule	2.3853828	1.2542208	0.010655
CD8A	CD8a molecule	2.2957113	1.1989412	0.0344056
LY9	lymphocyte antigen 9	2.3601296	1.2388661	0.0143012
Other DEGs related to adaptive immunity				
VPREB3	V-set pre-B cell surrogate light chain 3	15.36314	3.9414012	0.007275
LAX1	lymphocyte transmembrane adaptor 1	2.3150202	1.2110248	0.009064
BLK	BLK proto-oncogene, Src family tyrosine kinase	5.8224101	2.5416164	0.0271083
MSC	musculin	3.3339612	1.7372373	0.0444498
HSH2D	hematopoietic SH2 domain containing	2.4794627	1.3100276	0.0202166

Also, the transcripts of some cytokines, chemokines and their receptors were increased, which could activate host immune defense. The up-regulated chemokines included CCL11 (C-C Motif Chemokine Ligand 11) for eosinophils, CXCL13 (C-X-C Motif Chemokine Ligand 13) for B-lymphocytes, CCL19 (C-C Motif Chemokine Ligand 19) for T-lymphocytes and B-lymphocytes and its receptor CCR7. Likewise, the expression of some cytokine receptors was increased, such as interleukin receptors (IL2RA and IL17REL) and tumor necrosis factor receptor superfamily (TNFRSF7, TNFRSF9, TNFRSF13C and TNFRSF17). We also found interferon-related genes significantly up-regulated, including IRF4 (Interferon Regulatory Factor 4), IFITM10 (Interferon Induced Transmembrane Protein 10), IFRD1 (Interferon Related Developmental Regulator 1) and SYNDIG1 (Synapse Differentiation Inducing 1). Besides, the transcript expression of MMP9, a member of matrix metallopeptidase, was increased as well. VMP1 (Vacuole Membrane Protein 1) and RUFY4 (RUN and FYVE domain containing 4), which involved in autophagy, were also up-regulated (**Table 2**).

In addition, the abundance of transcripts related to Fc receptors (FCRL2, FCRL4, FCRL5, FCRLA and FCMR) and immunoglobulin superfamily were also found increased. The up-regulated immunoglobulin superfamily members included IGDCC4 (Immunoglobulin Superfamily DCC Subclass Member 4), SIRPG (Signal Regulatory Protein Gamma), SLAMF6 (SLAM Family Member 6), CTLA4 (Cytotoxic T-lymphocyte Associated Protein 4), JCHAIN (Joining Chain Of Multimeric IgA and IgM) and IGLL5 (Immunoglobulin Lambda Like Polypeptide 5). What’s more, there are other up-regulated genes encoding cluster of differentiation, including CD20, CD22, CD72, CD79A for B cells and CD8A, CD28, CD229 for T cells. Several genes involved in regulating lymphocytes were also up-regulated, including VPREB3 (V-set Pre-B Cell Surrogate Light Chain 3), LAX1 (Lymphocyte Transmembrane Adaptor 1), BLK (BLK Proto-Oncogene, Src Family Tyrosine Kinase), MSC (Musculin) and HSH2D (Hematopoietic SH2 Domain Containing) (**Table 2**). Taken together, these DEGs indicated the activation of adaptive immunity during pulmonary mucormycosis.

Beyond these DEGs, pulmonary mucormycosis also significantly induced many other genes related to immune response (**Supplementary Table S3**). Numerous genes involved in infection response and immune defense exhibited up-regulated expression, indicating the host tried to activate immune reactions upon pathogen challenge.

**Figure 2:**
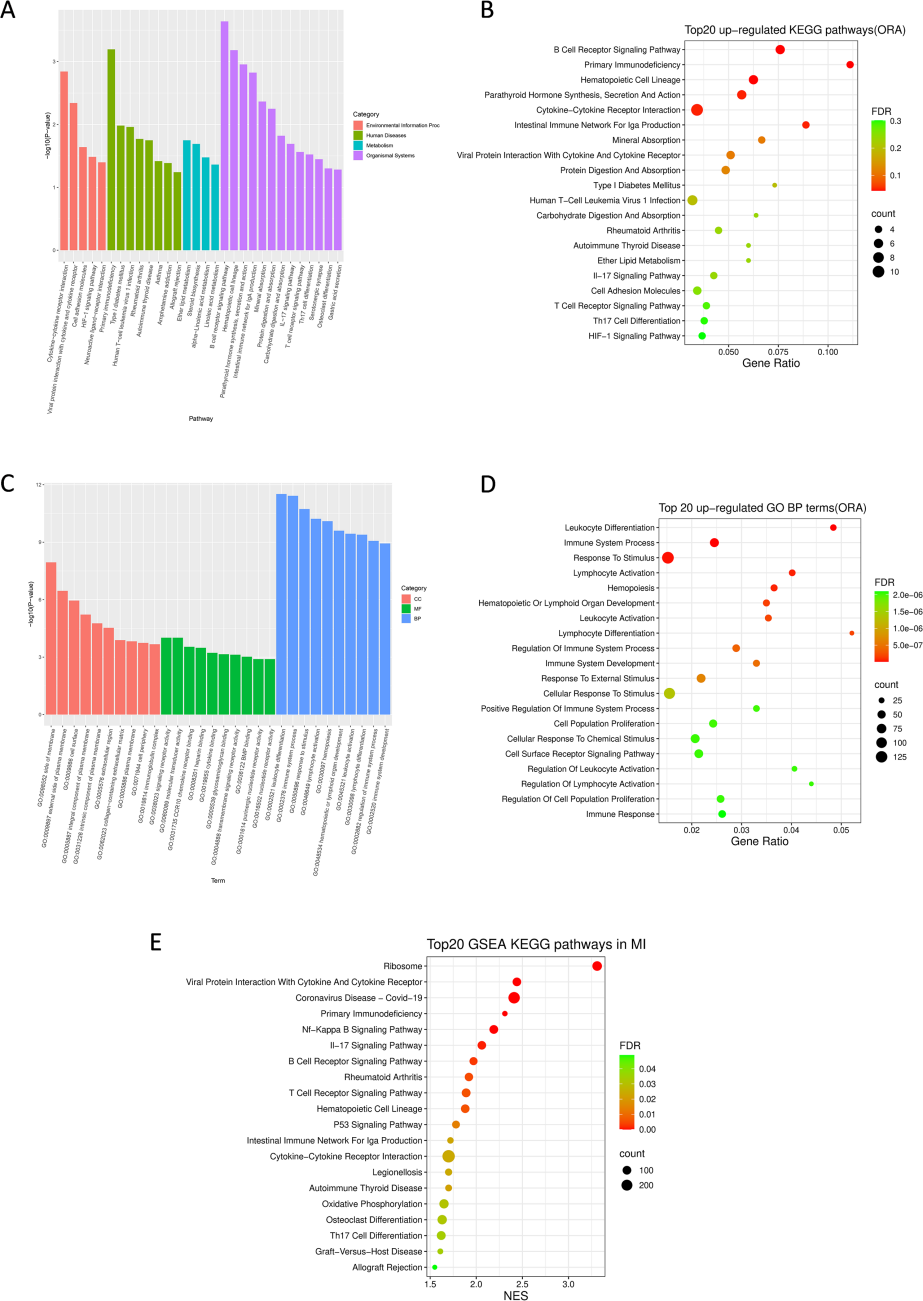
KEGG and GO enrichment analysis of up-regulated DEGs in pulmonary mucormycosis. **(A)** KEGG analysis of up-regulated pathways in pulmonary mucormycosis. These pathways are classified into 4 categories, including environmental information processing, human diseases, metabolism and organismal systems. **(B)** Bubble plot of top 20 up-regulated KEGG pathways(ORA) in pulmonary mucormycosis. The bubble size represents the number of genes enriched in the pathway and the color intensity of the bubbles represents the enrichment FDR of the pathway. **(C)** GO analysis of up-regulated terms in pulmonary mucormycosis. These terms include 3 categories: biological processes, molecular functions and cellular component. **(D)** Bubble plot of top 20 up-regulated GO biological processes (ORA) in pulmonary mucormycosis. The bubble size represents the number of genes enriched and the color intensity of the bubbles represents the enrichment FDR. **(E)** Bubble plot of top 20 up-regulated KEGG pathways in GSEA. The bubble size represents the number of genes enriched and the color intensity of the bubbles represents the enrichment FDR.

To characterize the functions of these DEGs, we performed KEGG and GO enrichment analysis. Compared to the control group, KEGG analysis showed a total of 28 up-regulated pathways significantly enriched in infected lung tissues (**Supplementary Table S4**). Among them, several immune-related pathways were activated, including B cell receptor signaling pathway, hematopoietic cell lineage, cytokine-cytokine receptor interaction, IL-17 signaling pathway, cell adhesion molecules, T cell receptor signaling pathway and Th17 cell differentiation, illustrating strong inflammatory response during pulmonary mucormycosis (**Figures 2A, B**; **Supplementary Table S4**). Notably, primary immunodeficiency and type I diabetes mellitus pathways were enriched, reflecting impaired host immune function in infected lungs, which might be responsible for susceptibility to mucormycosis (**Figures 2A, B**; **Supplementary Table S4**). In addition, the host immune response resembled other infections or inflammatory diseases such as intestinal immune network for IgA production, viral protein interaction with cytokine and cytokine receptor, human T-cell leukemia virus 1 infection, rheumatoid arthritis and autoimmune thyroid disease (**Figure 2A**; **Supplementary Table S4**). Also, HIF-1 signaling pathway was induced due to lung injury caused by fungal invasion (**Figure 2A**; **Supplementary Table S4**).

GO analysis was used to annotate gene product functions from three aspects (biological processes, molecular functions, and cellular component). Similarly, GO analysis revealed that a lot of immune-related biological processes were up-regulated in pulmonary mucormycosis (**Figures 2C, D**; **Supplementary Table S5**). Among the top-20 significantly up-regulated terms, more than half were associated with host immune response, including leukocyte differentiation, immune system process, lymphocyte activation, leukocyte activation, lymphocyte differentiation, regulation of immune system process, immune system development, positive regulation of immune system process, regulation of leukocyte activation, regulation of lymphocyte activation and immune response, suggesting that both innate and adaptive immunity were activated during pulmonary mucormycosis (**Figure 2D**). Besides, there were 4 pathways related to response to stimulus (**Figure 2D**). The other 5 pathways were also indirectly related to the immune response (**Figure 2D**). These enriched GO terms presented a strong inflammatory response induced in pulmonary mucormycosis.

The above enrichment analyses used an exploratory DEG definition based on raw p value < 0.05 and |log2FoldChange| > 1, which may introduce false positives. Consistent with this, only a limited number of KEGG pathways remained significant after multiple-testing correction in the ORA results. Therefore, we further performed GSEA to evaluate transcriptomic pathway changes, which base on interrogates genome-wide expression differences between the two groups. Pathways were considered significant up-regulated at FDR q < 0.25 and NES > 1 (**Supplementary Table S15**) and the top 20 enriched pathways were shown in **Figure 2E** (all with FDR < 0.05). Notably, the top 20 enriched KEGG pathways were highly concordant between ORA and GSEA, including enrichment of B cell receptor signaling, T cell receptor signaling, IL-17 signaling/Th17 cell differentiation, cytokine–cytokine receptor interaction and hematopoietic cell lineage, as well as pathways related to infection and inflammatory diseases. In addition, primary immunodeficiency was also enriched in GSEA, indicating a potential impairment of host immune status. Moreover, GSEA captured broader immune signaling modules (e.g., NF-κB, TNF, Nod-like receptor, C-type lectin receptor, chemokine signaling, neutrophil extracellular trap formation, NK cell mediated cytotoxicity and Th1/Th2 differentiation). Collectively, these consistent findings supported a broad transcriptional activation of host immune response in pulmonary mucormycosis.

In summary, the results of transcriptomics analysis revealed a widespread upregulation of genes and pathways involved in immune and inflammatory responses, indicating the host attempted to modulate defensive mechanisms against pathogen challenge.

### Proteomic profiling of pulmonary mucormycosis revealed impaired immune function

**Figure 3:**
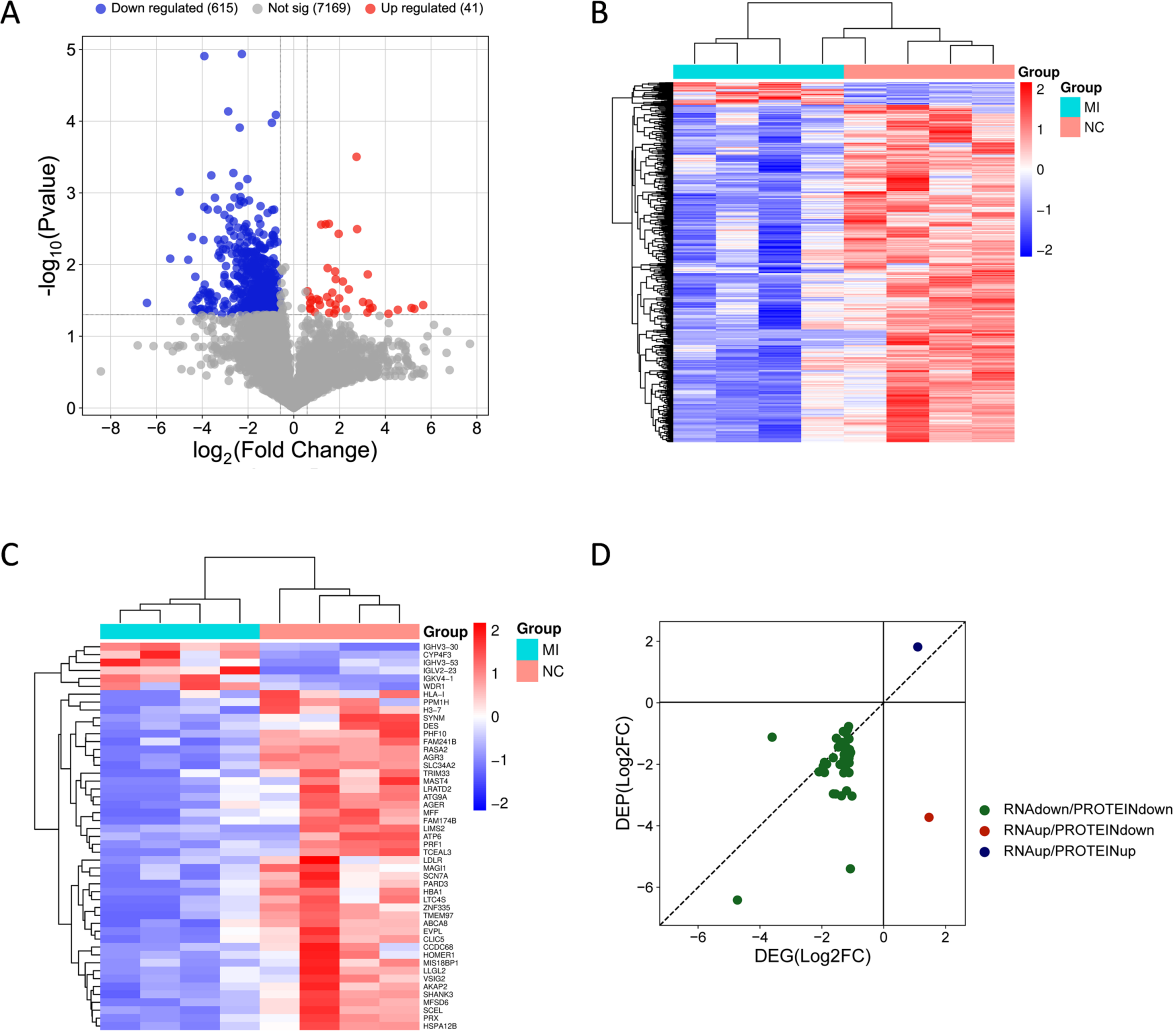
Proteomic profiling of pulmonary mucormycosis. **(A)**Volcano plots of proteins in infected lung tissues and non-infected controls. The proteins with a p value < 0.05 and |log2FoldChange| > 0.585 were considered DEPs. Red and blue dots indicate up-regulated and down-regulated proteins, respectively. Gray dots indicate the non-significant proteins. **(B, C)** Heatmap of all DEPs **(B)** and top 50 most significantly DEPs **(C)** distribution. Columns represent the samples and groups and rows represent the DEPs. The red and blue proteins represent a higher and a lower expression, respectively. The color intensity (blue to red) corresponds to their expression Z-scores. **(D)** Scatter plot of overlapping DEGs and DEPs. The x-axis and y-axis represent the log2FC of DEGs and DEPs, respectively. Green dots indicate simultaneous down-regulation of RNA and protein levels, blue dots represent simultaneous up-regulation, and red dots represent up-regulation of RNA levels but down-regulation of protein levels.

We conducted data-independent acquisition proteomic profiling of four lung tissues from three pulmonary mucormycosis patients. After comparing the infected group with the control group, a total of 656 proteins were found differently expressed (p value < 0.05 and |log2FoldChange| > 0.585), including 41 up-regulated proteins and 615 down-regulated proteins (**Figure 3A**; **Supplementary Tables S6**, **S7**). Hierarchical clustering analysis based on all DEPs (**Figure 3B**) and the top 50 most significantly altered DEPs (**Figure 3C**) demonstrated different proteomic alterations in mucormycosis. Different from transcriptome, most of DEPs were down-regulated (**Figures 3A–C**, **Supplementary Tables S6**, **S7**). To further evaluate the concordance between transcriptomic and proteomic changes, we compared overlapping DEGs and DEPs in **Figure 3D** (**Supplementary Table S12**). The majority of overlapping gene–protein pairs exhibited concordant down-regulation (**Figure 3D**).

It is worth noting that, contrary to the results of transcriptome, only a few proteins involved in immune response had significantly increased abundance (**Table 3**). Some of these proteins might be beneficial for host defense, such as rho guanine nucleotide exchange factor 1, BPI fold-containing family B member 1, copper-transporting ATPase 1, WD repeat domain 1, integrin beta-2, immunoglobulin heavy variable 6-1, neutrophil cytosol factor 4, IGL@ protein and mucin 5AC, oligomeric mucus/gel-forming. Conversely, the increased expression of docking protein 3, which is an adaptor molecule and belongs to the Dok family, was detrimental to antifungal immunity (24, 25). Notably, about half of the up-regulated proteins were fragments of immunoglobulins, indicating that humoral immunity might play a role in immune response against fungal pathogens (**Supplementary Table S6**). Nevertheless, the abundance of numerous proteins was reduced, spanning diverse functions, indicating compromised host physiological functions under pathogen challenge, particularly immune defense (**Supplementary Table S7**). These findings suggested that suppression of immune-related proteins might contribute to impaired pathogen clearance during mucormycosis.

**Table 3:** Up-regulated DEPs associated with host immunity.

Protein	Gene	Foldchange (MI/NC)	log2FC	P-value
Rho guanine nucleotide exchange factor 1	ARHGEF1	2.896	1.533927	0.002696
BPI fold-containing family B member 1	BPIFB1	2.292	1.196382	0.002774
Copper-transporting ATPase 1	ATP7A	6.813	2.768222	0.003203
N(4)-(beta-N-acetylglucosaminyl)-L-asparaginase	AGA	2.785	1.477912	0.011203
Cytochrome P450 4F3	CYP4F3	9.395	3.231838	0.013718
WD repeat domain 1, isoform CRA_a (Fragment)	WDR1	5.293	2.403958	0.022194
Integrin beta-2	ITGB2	1.621	0.696786	0.02796
Immunoglobulin heavy variable 6-1	IGHV6-1	1.999	0.999466	0.030269
Junctional adhesion molecule-like	JAML	3.005	1.587564	0.034186
Neutrophil cytosol factor 4	NCF4	1.643	0.7163	0.034473
Platelet-activating factor acetylhydrolase	PLA2G7	3.523	1.816873	0.036135
IGL@ protein	IGL	50.36	5.654152	0.036648
Apoptotic protease-activating factor 1	APAF1	1.644	0.717081	0.040278
Mucin 5AC, oligomeric mucus/gel-forming	MUC5AC	10.05	3.329824	0.041084
Docking protein 3	DOK3	1.659	0.730684	0.041842

**Figure 4:**
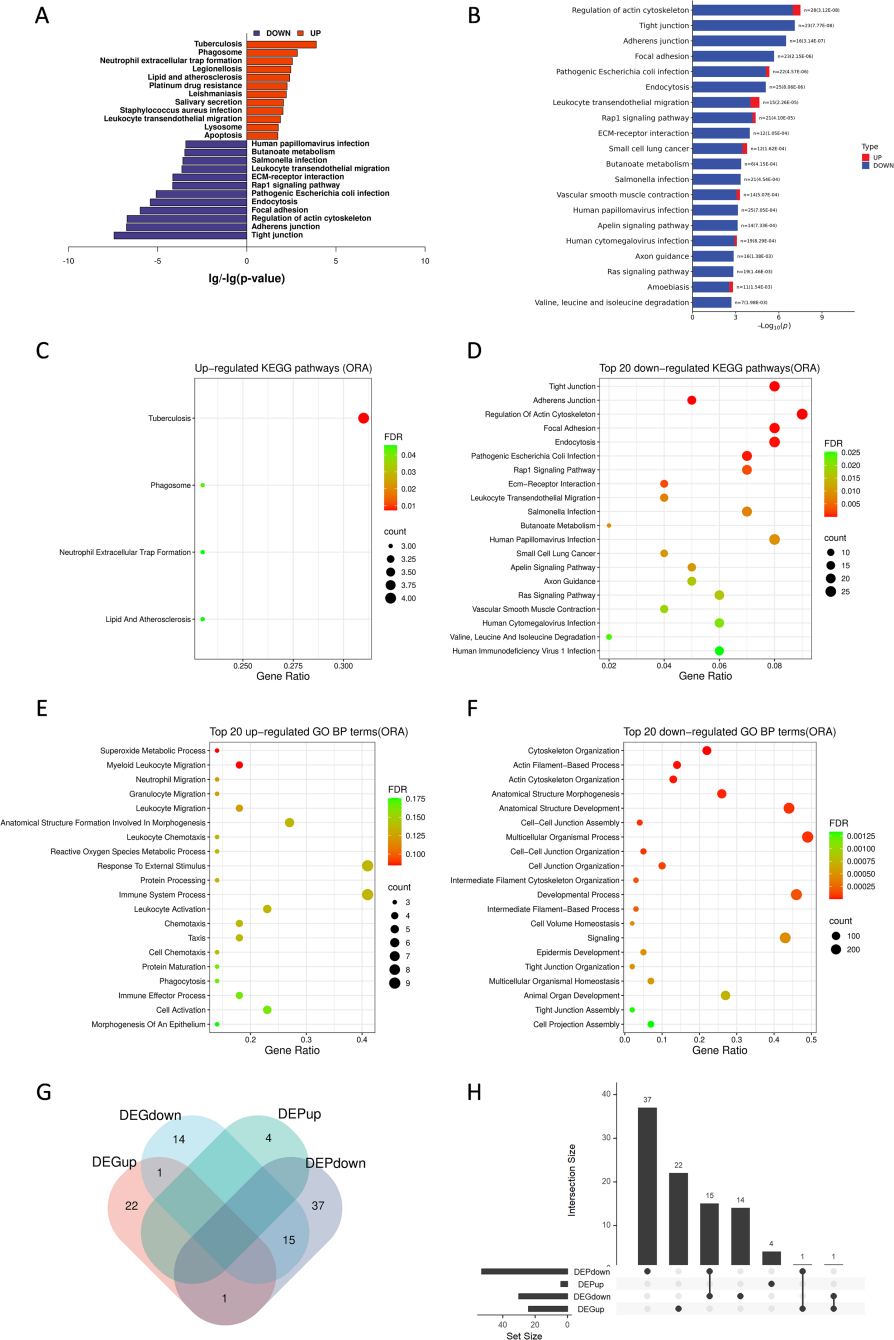
KEGG and GO enrichment analysis of DEPs in pulmonary mucormycosis. **(A)** Butterfly plot of the top 12 significant KEGG pathways enriched from up-regulated DEPs and down-regulated DEPs. The x-axis represents log_10_(p-value) or -log_10_(p-value). Blue and red bars represent pathways enriched from down-regulated and up-regulated proteins, respectively. **(B)** KEGG enrichment analysis of the top 20 most significant pathways in pulmonary mucormycosis. The x-axis represents -log_10_(p-value) and the y-axis displays pathway names. The proportion of upregulated (red) and downregulated (blue) proteins in each pathway is shown in color-coded bars. Bar labels indicate the total protein counts and p-value. **(C–E)** Bubble plot of **(C)** 4 up-regulated KEGG pathways and **(D)** top 20 down-regulated KEGG pathways in pulmonary mucormycosis. Bubble plot of **(E)** top 20 up-regulated GO biological processes and **(F)** top 20 down-regulated GO biological processes in pulmonary mucormycosis. The bubble size represents the number of proteins enriched in the pathway and the color intensity of the bubbles represents the enrichment FDR. **(G)** Venn diagram of KEGG pathways uniquely or commonly enriched by transcriptomic and proteomic analysis. KEGG pathways were compared using a four-set Venn diagram. Numbers indicate the count of pathways. **(H)** Upset plot of KEGG pathways uniquely or commonly enriched by transcriptomic and proteomic analysis. The horizontal bars and vertical bars represent the total number of pathways in each category (set size) and the number of pathways in each specific intersection (intersection size), respectively. Connected black dots indicate the combinations of datasets.

Then we performed KEGG and GO enrichment analysis to investigate the function of DEPs. KEGG analysis demonstrated that there were more down-regulated pathways than up-regulated pathways and many immune-related pathways were down-regulated overall, indicating that host functions, especially immune response in pulmonary mucormycosis were impaired in protein level (**Figures 4A, B**; **Supplementary Tables S8**, **S9**). Among the limited up-regulated enrichment pathways, phagosome and neutrophil extracellular trap formation were associated with immune defense, likely reflecting that the host still had some immune defense functions but it was not enough to resist infection (**Figure 4C**; **Supplementary Table S8**). The top 20 most significantly down-regulated pathways included a subset associated with immune defense and infection, such as endocytosis, leukocyte transendothelial migration and some infectious diseases, indicating dysfunction of the host immune system. Regulation of actin cytoskeleton, which might modulate cell migration and phagocytosis, was also down-regulated significantly. Moreover, we observed there were several pathways associated with cell junctions and adhesion, including tight junction, adherens junction, focal adhesion, Rap1 signaling pathway and ECM-receptor interaction, suggesting the immune barrier function of the lungs might be damage under fungal invasion (**Figure 4D**). Besides, several pathways associated with immune defense were also significantly down-regulated, such as chemokine signaling pathway, apoptosis, autophagy, peroxisome and PRR signaling pathways, including C-type lectin receptors, NOD-like receptors and RIG-I-like receptors (**Supplementary Table S9**). Collectively, these results revealed impaired immune function in the infection foci of pulmonary mucormycosis.

The results of GO enrichment analysis were similar to KEGG analysis. We observed more GO terms including immune-related terms were down-regulated (**Supplementary Tables S10**, **S11**). Among the 20 most significantly up-regulated biological processes, half were associated with host immune response, including innate immune response in mucosa, myeloid leukocyte migration, organ or tissue specific immune response, mucosal immune response, neutrophil migration, granulocyte migration, neutrophil mediated immunity, neutrophil activation, leukocyte migration, granulocyte activation (**Figure 4E**). However, the FDR of all these pathways were not < 0.05, indicating that the host immune function was limited. These enriched terms confirmed that innate immunity was critical in the defense against fungal infection. Similar to KEGG, we found several GO terms associated with cell junction and cytoskeletal regulation among the top 20 most significantly down-regulated biological processes. The former included cell-cell junction assembly, cell-cell junction organization, cell junction organization, tight junction organization and tight junction assembly, suggesting that the defense barriers against microbial invasion had been disrupted (**Figure 4F**). The downregulation of cytoskeleton organization, actin filament-based process, actin cytoskeleton organization and cell projection assembly indicated potential dysfunction of cellular phagocytic function. Additionally, numerous biological process terms associated with T cell immunity were significantly down-regulated, indicating severe impairment of adaptive immune function (**Supplementary Table S11**). These results were consistent with the refractory fungal infections observed.

To compare alterations of enriched pathways between transcriptomic and proteomic profiling, we performed an overlap analysis of KEGG pathways enriched from DEGs and DEPs and generated a Venn plot (**Figure 4G**) as well as an UpSet plot (**Figure 4H**) (**Supplementary Tables S4**, **S8**, **S9**, **S13**, **S14**). We observed that there were few common up-regulated pathways, while most of the immune-related pathways were only up-regulated in DEGs enriched pathways (**Figures 4G, H**) (**Supplementary Table S14**). In contrast, we found that most of the overlapping pathways were down-regulated simultaneously, such as tight junction, ECM–receptor interaction, focal adhesion, PI3K–Akt signaling, Hippo signaling and Ras signaling pathways, suggesting a severe damage of lung tissue structure and barrier function during pulmonary mucormycosis (**Figures 4G, H**) (**Supplementary Table S14**).

In conclusion, the proteomic signature of pulmonary mucormycosis indicated that immune function against fungal infection was impaired, which might result in high mortality rates and difficulty in treatment.

Taken together, transcriptomic analysis showed upregulation of diverse immune-related genes, suggesting the host attempted to activate signaling pathways involved in immune defense against mucormycosis. However, proteomic profiling demonstrated impaired host immune function and barrier integrity at the protein level. These results revealed a failed activation of host immune response.

## Discussion

In this study, we described transcriptome and proteome of pulmonary mucormycosis patients to explore host immune landscape for the first time. Previous studies mainly focused on the transcriptome profiles of COVID-19-associated mucormycosis patients as well as cellular and animal models of mucormycosis. To date, there is a lack of proteomic investigations on mucormycosis. Our results highlighted the immune response in infected lung tissues intuitively and provided a new perspective for the pathogenesis of pulmonary mucormycosis.

It is well known that diabetes mellitus constitutes an important risk factor for susceptibility to mucormycosis (26). All five patients enrolled in this study had diabetes mellitus and two patients had ketoacidosis, which might be a potential cause for their infection. Hyperglycemia induces upregulation of glucose-regulated protein 78 (GRP78), advanced glycation end-products (AGE) and availability of iron as well as impairs phagocytic capacity and other microbicidal activity of immune cells (27–30). In this study, we employed a self-paired control design to explore immune mechanisms of pulmonary mucormycosis beyond established risk factor. Furthermore, the KEGG pathway of primary immunodeficiency was enriched among the infected group (**Figures 2B, E**; **Supplementary Tables S4**, **S15**), suggesting that in addition to diabetes, there may be underlying immunologic factors due to pathogen infection that impair the immune response. The underlying mechanisms warrant further investigation, as such immunopathogenic effects potentially contribute to the lack of clinical improvement in certain patient populations.

A notable finding of our study was the discordance between transcriptomic and proteomic immune responses in pulmonary mucormycosis. It was revealed that transcriptomics analysis showed activation of host immune defense function at the transcriptional level, whereas proteomic profiling suggested there were deficiencies in host immune response against infection at the protein level. These results are consistent with previous researches on mucormycosis. A study on CAM patients reported deficient phagocytosis in circulating monocytes with a significantly down-regulated of pathways related to endocytic pathways, phagosome maturation and the cytoskeletal regulation of phagocytosis (12). Similarly, another transcriptomic study demonstrated immune derangement in COVID-19 associated pulmonary mucormycosis (CAPM) patients (13). Similar phenomena were also observed in *in vitro* and *in vivo* models: *R. oryzae* infection induced a down-regulation of many PRRs genes and Toll-like receptor-signaling pathway in peripheral blood mononuclear cells (PBMCs) compared to *C. albicans* and *A. fumigatus* infection, and completely impaired the ability to generate reactive oxygen species (ROS) (15); in the drosophila melanogaster model, *Zygomycetes* infection also down-regulated several genes involved in pathogen recognition and immune defense compared to *Aspergillus* infection (16). Our results of proteomic profiling also revealed downregulation of many immune-related genes and pathways, collectively demonstrating compromised host immune defenses in mucormycosis. These results together explain the possible reason why mucormycosis is more aggressive and difficult to cure than other fungal infections.

Similar transcript–protein discordance has also been reported in some other multi-omics studies on infectious diseases. For instance, a tomato spotted wilt virus infection model in insect gut tissues showed discordance between DEGs and DEPs, indicating distinct and dynamic regulatory mechanisms of transcript and protein abundance (31). In human infection contexts, DEGs and DEPs exhibited opposing regulatory patterns in immunocompromised populations following SARS-CoV-2 infection; the host responses in SARS-CoV-2-infected lung epithelial cells also revealed overlapping yet distinct immune signatures across the transcriptome and proteome (32, 33). In addition, complex host immune dysregulation reflected by transcriptomic and proteomic signatures was also reported in children with severe malarial anemia (34). Mechanistically, although RNA-seq is sensitive for transcriptional activation, protein abundance is regulated not only by transcription but also by post-transcriptional, translational and protein degradation regulation (35). For example, in a multi-omics study on macrophage activation model, protein secretion could be independent of transcription due to processes such as ectodomain shedding and proteolytic processing, leading to the protein-level responses might lag behind or diverge from transcript-level changes (36).

In our study, this transcript–protein discordance was further supported through integrative analyses that transcriptomic and proteomic findings were not completely contradictory but rather exhibited a partial discordance (**Figures 3D**, **4G, H**; **Supplementary Tables S12**, **S14**). On the transcriptomic layer, broad induction of immune-defense programs was observed, including iron-homeostasis mediators (e.g., HAMP, HP, TF/TP, LTF) and multiple pathogen-sensing/immune-regulatory genes (e.g., PRR, cytokines, chemokines), along with pathways related to innate and adaptive immunity. In contrast, proteomic profiling showed mainly down-regulation of immune- and barrier-related pathways. Moreover, integrative overlap analyses indicated the shared down-regulated signals were related to structural and signaling modules (e.g., tight junction, focal adhesion, and ECM–receptor interaction), suggesting lung tissue damage and compromised barrier integrity during pulmonary mucormycosis. Collectively, these results suggested that although transcriptomic profiling indicated an attempted immune activation, the protein-level execution of host defense might be constrained in severely infected lung tissues, consistent with compromised barrier integrity and impaired immune responses during pulmonary mucormycosis.

In mucormycosis, severe tissue destruction, hypoxia, metabolic dysregulation and fungal immune-modulatory strategies may further compromise protein synthesis or stability in infected lung tissues (37, 38). The possible mechanisms include severe tissue destruction and disrupted cellular composition at infection sites, strong post-transcriptional/translational regulatory constraints during overwhelming infection and enhanced protein degradation. Collectively, these mechanisms could contribute to activated transcriptome but insufficient effector proteome, which was consistent with impaired host defense during pulmonary mucormycosis. Therefore, we speculated that during pulmonary mucormycosis, transcriptional upregulation might reflect an attempted or compensatory host response, but failed to translate into effective protein-level responses, which were required for effective host defense against mucormycosis. Given that the patients in our study required surgical intervention after failure of systemic antifungal therapy, the observed suppression of immune-related proteins could reflect the pathophysiological state of pulmonary mucormycosis. The failure to increase expression of functional immune-related proteins might contribute to a critical mechanism underlying disease severity and poor clinical outcomes.

Different from proteomic findings, our transcriptomic profiling showed a broad activation of immune-related genes and pathways. For instance, cytokine/chemokine-associated signaling was induced; pathogen-sensing and innate immune regulation were activated, such as increased transcripts of PRRs. Also, adaptive immune activation was reflected by enrichment of B- and T-cell related pathways, together with upregulation of some lymphocyte markers and regulators. Notably, iron-homeostasis emerged as an important signature, exemplified by marked upregulation of HAMP along with HP, TF/TP and LTF. Collectively, these transcriptomic findings indicated a broad “attempted” immune activation program in infected lung tissues at the mRNA level, which provides an important context for interpreting the limited or suppressed immune-protein responses observed by proteomics. These results were consistent with studies focusing on transcriptome of macrophages, epithelial cells as well as murine and zebrafish infection models (14, 18–21). While a subset of these up-regulated genes and pathways contributes to antifungal immunity, the functions and mechanisms of many remain uncharacterized.

Pulmonary mucormycosis is initiated by inhalation of Mucorales spores. In the healthy host, the first-line immune barrier relies on airway/alveolar epithelial cells, resident alveolar macrophages, recruitment of neutrophils as well as cytokine/chemokine production that modulates innate immune functions and subsequent adaptive immune responses (1, 9). As we all know, pulmonary mucormycosis is an opportunistic mycosis that infects hosts with qualitative or quantitative defects in immunity, including patients with severe neutropenia, recipients of corticosteroids or other immunosuppressive medications, poorly controlled diabetes mellitus and those with iron overload states (39). Consequently, therapeutic strategies aimed at reversing host immune compromise could potentially improve survival outcomes of patients with severe infections.

Iron homeostasis plays an important role in antifungal immunity, particularly in mucormycosis (40–42). Prior studies have reported serum unbound iron is a critical factor for mucormycosis susceptibility in diabetic ketoacidosis (DKA) patients and limiting iron utilization by fungus is beneficial for host defense (14, 30, 43). Our transcriptomics analysis found several DEGs (HAMP, HP, TP and LTF) involved in iron homeostasis was up-regulated. HAMP was the most significantly up-regulated DEGs, which encode hepcidin and play a central role in iron metabolism. Similarly, a previous study has found iron-related genes were differentially expressed in infected macrophages and the association between HAMP gene polymorphisms and CAPM susceptibility has been investigated (14, 44). Hepcidin was also strongly induced in murine models of *Candida* and *Aspergillus* infection and it contributed to host defense against *Candida albicans* infection (45, 46). Haptoglobin encoded by HP, transferrin encoded by TP and lactoferrin encoded by LTF, can also restrict iron acquisition by fungal pathogens. Haptoglobin is up-regulated in paracoccidioidomycosis, aspergillosis and fusarium keratitis as well (47–49). Transferrin has antimicrobial activity against several bacterial and fungal pathogens, such as *Candida albicans* and *Histoplasma capsulatum (*50–53). Lactoferrin has already been found antifungal effects against mucor species in combination with Amphotericin B (54, 55). The precise functional role of these iron-related proteins in mucormycosis and other fungal infection diseases needs further exploration. Our results indicated a discordance between transcriptional upregulation and protein expression of these iron-metabolism proteins and supplementing these antibacterial-active proteins may constitute a novel adjunctive antifungal strategy (56).

Recognition of fungal pathogens by PRRs is critical in host immunity. Previous research has identified altered expression of dectin-1, TLR2, and TLR4 in circulating monocytes of mucormycosis patients and demonstrated that TLRs were essential for pro-inflammatory responses against mucor species (57–59). Our results showed elevated transcripts of four PRRs (TLR10, ZBP1, CLEC17A and AIM2). The genetic polymorphism of TLR10 was associated with susceptibility to aspergillosis (60, 61). ZBP1 was necessary for inflammatory PANoptosis induced by *C. albicans* and *A. fumigatus* infection (62, 63). CLEC17A, a member of C-type lectin receptor (CLR) family, might be capable of recognizing fungal β-glucans, but its role in fungal infections remains uninvestigated (64). AIM2 was beneficial for host defense against *Aspergillus* infection but enhanced *Candida* infection (65, 66). It was also up-regulated in *Malassezia* folliculitis (67). In mucormycosis, the functions of these PRRs remain unknown and warrant further mechanistic investigation.

Cytokines and chemokines promote inflammatory response and recruitment of immune cells during infection. Cytokine-cytokine receptor interaction was significantly enriched in our KEGG analysis (**Figures 2A, B**). GO analysis also showed a number of terms related to cytokines enriched, including cytokine-mediated signaling pathway, regulation of cytokine production, cytokine production, positive regulation of cytokine production, cellular response to cytokine stimulus, response to cytokine, cytokine-mediated signaling pathway (**Supplementary Table S5**). Previous studies have found a few cytokines and chemokines induced during mucormycosis infection, such as TNF-α, interferon-γ, GM-CSF, interleukins, and so on (37, 68–70). Our data found elevated expression of several transcripts in chemokine family, including CCL11, CXCL13, CCL19 and its cognate receptor CCR7, which contribute to the recruitment of eosinophils and lymphocytes. CCL11(eotaxin) serves as a major chemoattractant for eosinophils and Th2 cells. It could be induced by *A. fumigatus*, which was associated with allergic airway responses and probably participated in the pathogenesis of allergic bronchopulmonary aspergillosis (71–73). Notably, some case reports have documented allergic-like manifestations in mucormycosis, but the underlying mechanisms are unknown (74–78). The expression of CXCL13 was also up-regulated in *Pneumocystis* and *Candida albicans* infection, though its precise function remains incompletely understood (79, 80). CCL19 expression was elevated during *Aspergillus* infection and its receptor CCR7 was also up-regulated in *Aspergillus* and *Paraccocidium brasiliensis* infection; however, CCR7 deficiency reduced host susceptibility to aspergillosis (81–89). In summary, the functions of these chemokines remain not fully defined in mucormycosis.

Beyond chemokines, transcriptional upregulation was observed in some members of interleukin receptor family (IL2RA and IL17REL) and TNF receptor superfamily (TNFRSF7, TNFRSF9, TNFRSF13C and TNFRSF17). Current studies have documented IL-2 and IL-17 up-regulated during mucormycosis and they strengthened host defense (90–92). For instance, IL-2-stimulated natural killer (NK) cells could damage mucormycetes (92). In murine mucormycosis model, IL-17 signaling contributed to pathogen clearance and antifungal immunity. Concordantly, our KEGG enrichment analysis also found significant upregulation of IL-17 signaling pathway (**Figures 2A, B**). TNFRSF regulates a spectrum of immune cell responses. TNFRSF9 (CD137) and TNFRSF13C (BAFFR) exerted protective roles in Candidiasis and *Pneumocystis* pneumonia, respectively (93–95). Moreover, our analysis showed some interferon-related genes (IRF4, IFITM10, IFRD1 and SYNDIG1) significantly up-regulated. Interferon-γ has been found to improve fungal clearance *in vitro* and murine models of mucor infection and a lot of clinical case reports have documented interferon as adjunctive antifungal therapy in mucormycosis (69, 96–107). In summary, the mechanisms by which these cytokines regulate anti-fungal immune responses in mucormycosis remain incompletely understood. Given our proteomic findings demonstrating compromised host immune function at infection sites, supplementation of effective cytokines may constitute a potential therapeutic strategy to enhance host immunity against fungal infection. Consequently, further research is warranted to develop novel adjunctive immunotherapeutic approaches.

Currently, whether adaptive immunity plays a critical role in mucormycosis remains uncertain. Our data showed a number of up-regulated DEGs and enriched pathways related to adaptive immune activation (**Table 2**; **Figure 2**). For instance, T cell receptor signaling pathway was enriched in KEGG analysis (**Figures 2A, B**). Notably, the expression of CTLA4, a coinhibitory receptor for T cell activation, was also up-regulated in other fungal infections, including paracoccidioidomycosis, candidiasis and *Lacazia loboi* infection (108–112). Blocking CTLA-4 confered protective effects in *Paracoccidioides brasiliensis* and *Cryptococcus neoformans* infections as well as fungal sepsis (*Candida* infection) (113–118). Similarly, blockade of PD-1/PD-L1 pathway demonstrated therapeutic efficacy in a murine model of pulmonary mucormycosis (119). These results suggest that immune checkpoint inhibitors (ICIs) could enhance T cell immune response to improve antifungal defense. CD28, a critical co-stimulatory molecule of T cells, is associated with diverse fungal infections. Low CD28^+^CD8^+^ T-cell counts were associated with mortality of invasive fungal infection, such as candidiasis and aspergillosis (120–122). *In vitro* studies demonstrated CD28 participated in immune responses against *Blastomyces dermatitidis*, *Pneumocystis* and *Paracoccidioides brasiliensis* infections (123–127). However, its role in mucormycosis remains unexplored. Moreover, our KEGG enrichment analysis also revealed Th17 cell differentiation pathway up-regulated (**Figures 2A, B**) and a study on CAM patients revealed that reduced Th17 cell frequency potentially correlated with susceptibility to mucormycosis (128). Our results of KEGG analysis showed B cell receptor signaling pathway was up-regulated as well. CD19 activate B cell immune response and modulate humoral immunity. However, CD19^+^ B cells were found reduced in CAM patients (129). Whether they exert antifungal roles requires further exploration.

In summary, our transcriptomic profiling revealed a broad upregulation of transcripts related to immune and inflammatory response, a substantial portion of which remain uncharacterized in fungal infections and need further investigation. Nevertheless, proteomic analysis demonstrated that the majority of proteins involved in immune defense failed to be induced. Only a limited subset of proteins was up-regulated, mainly enriched in innate immune pathways, such as phagosome, neutrophil extracellular trap formation and lysosome, suggesting their potentially critical roles in fungal clearance.

Because of the difficulty in obtaining lung specimens from healthy population, we took a self-controlled study design, which had intrinsic limitations and could not fully reflect normal pulmonary status. Due to pulmonary mucormycosis is a rare opportunistic infection, the number of cases that could be enrolled was inevitably limited. Our study only included surgical patients, who had severe disease manifestations and poor response to systemic antifungal therapy alone. Thus, our results could not represent the whole population of pulmonary mucormycosis. Additionally, the substantial clinical heterogeneity of mucormycosis should be considered, such as variations in infection sites and Mucorales species. Our study population had a single risk factor, diabetes mellitus, which further limited the generalizability of our results. Importantly, due to the limited availability of surgically resected human lung tissues, additional functional validation experiments were not feasible. Also, the limited sample size and heterogeneity of human surgical specimens reduced statistical power for single-gene discovery after multiple-testing correction. Therefore, raw p-value–based DEGs and enriched pathways were used for hypothesis-generating analyses, with FDR values provided as a sensitivity analysis. In conclusion, further studies with larger sample sizes, independent validation approaches and experimental models are warranted to identify the functions of DEGs and DEPs. Despite these limitations, our work provided deeper characterization of host immune response signatures during pulmonary mucormycosis, which may contribute to development of novel therapeutic strategies.

## Conclusion

Our study investigated the transcriptome and proteome of pulmonary mucormycosis for the first time. Transcriptomic profiling found upregulation of numerous genes and pathways related to immune defense, whereas proteomic analysis revealed dysfunction of antifungal immunity, collectively indicating a failed activation of host immune response in pulmonary mucormycosis.

## Data Availability

The datasets presented in this study can be found in online repositories. The names of the repository/repositories and accession number(s) can be found below: https://www.ebi.ac.uk/ena, PRJEB101444; http://www.proteomexchange.org/, IPX0013889000.
