# Supplementary figures and images for: Transcriptomic and proteomic profiling of pulmonary mucormycosis reveals a failed activation of host immune response
Source: Front Immunol. 2026 Feb 13;17:1732782. doi: 10.3389/fimmu.2026.1732782 (PMC12945811; doi:10.3389/fimmu.2026.1732782)

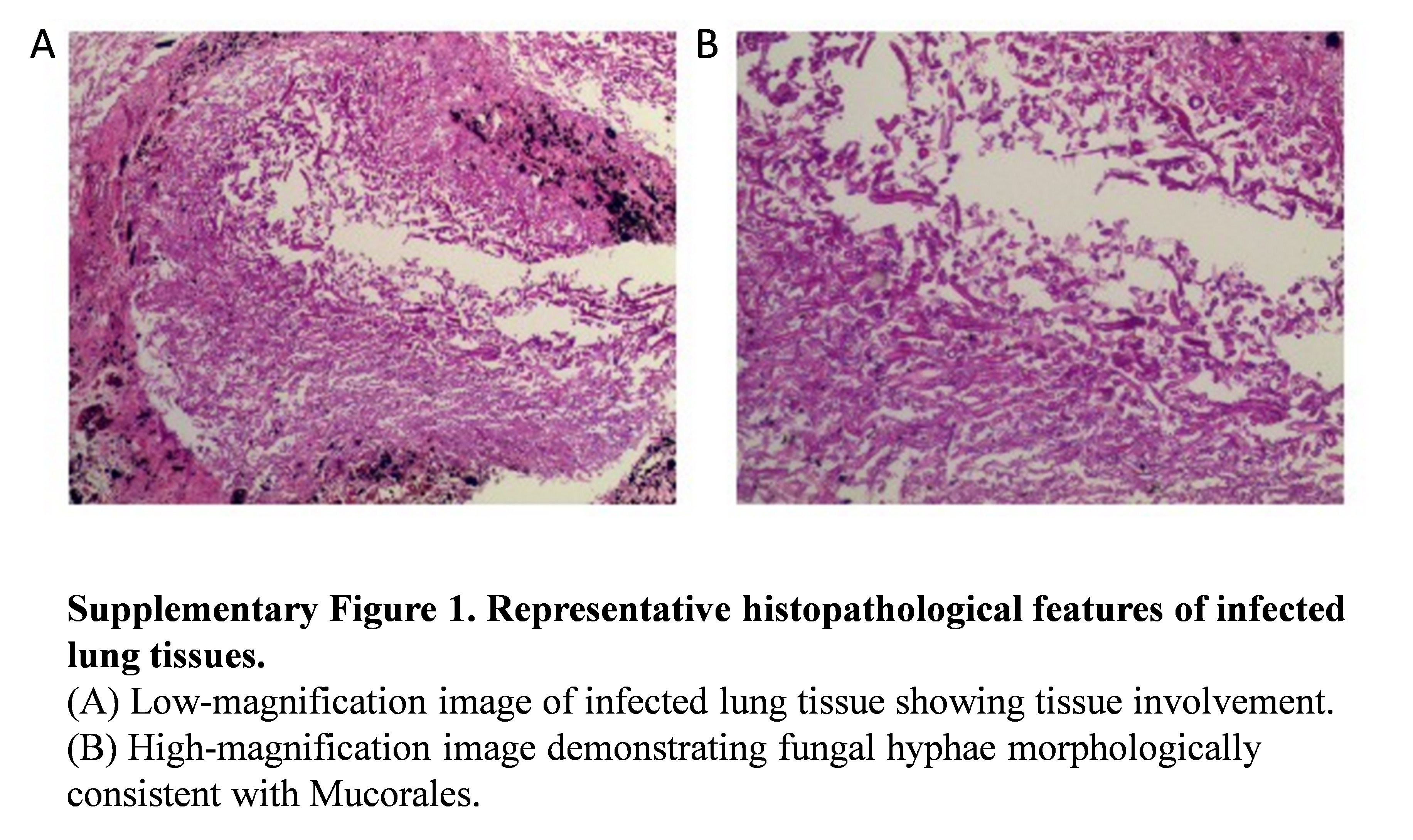

Supplement: Supplementary file 1 [file Image1.tif]

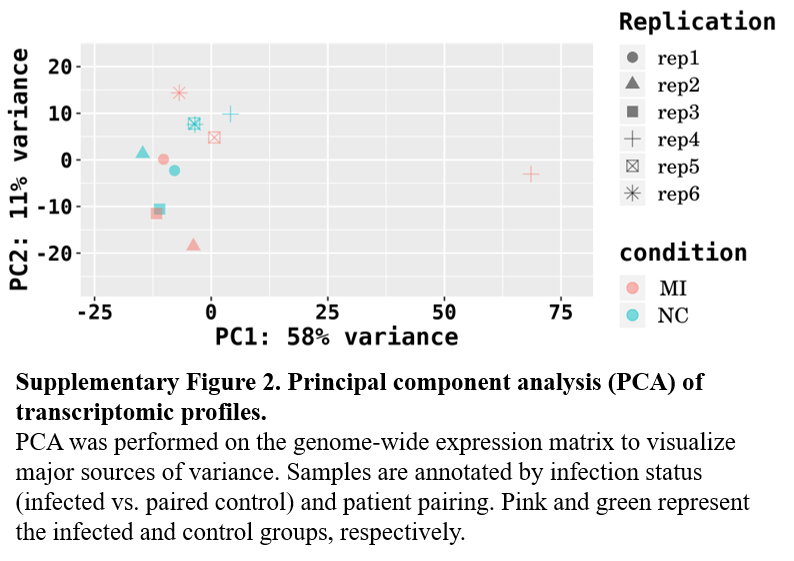

Supplement: Supplementary file 2 [file Image2.png]
